# Agentic AI in Dermatology: A Call to Action

**DOI:** 10.2196/94909

**Published:** 2026-06-05

**Authors:** Brian Chu, Heather Shen, Ivy Lee, Jules B Lipoff

**Affiliations:** 1 Department of Dermatology, Brown University, 593 Eddy St, APC 10th Floor, Providence, RI, 02903, United States, 1 4014447959; 2 Praxis Labs, Providence, RI, United States; 3 Comprehensive Dermatology Center of Pasadena, Pasadena, CA, United States; 4 Department of Dermatology, Lewis Katz School of Medicine, Temple University, Philadelphia, PA, United States

**Keywords:** agentic AI, AI agents, AI in dermatology, artificial intelligence, technology in dermatology

## Abstract

Artificial intelligence (AI) tools are shifting from passive, user-initiated tools to proactive *agentic AI* systems that are capable of autonomous, multi-step actions. These agents can independently gather information, execute sequential tasks, and collaborate with humans or other agents without requiring constant prompting from humans. Early adopters in health care have demonstrated early feasibility across multiple specialties and clinical settings. Dermatology is well-positioned to benefit given its high patient volumes, administrative burdens, and clinicopathological workflows. To guide responsible adoption of agentic AI, we propose a risk-stratification framework based on clinical risk and task reversibility. Barriers to widespread adoption of agentic AI include limitations in model reliability, interoperability across health records, and unresolved questions around liability, privacy, and regulation. Dermatologists must proactively engage via professional organizations and industry partnerships to ensure that agentic AI is developed safely, equitably, and in alignment with our values.

## From AI Tools to Agentic Teammates

Medicine is at an inflection point as artificial intelligence (AI) evolves from passive tools to proactive teammates, a concept known as *agentic AI* (throughout this article, the terms “agentic AI,” “AI agents,” and “agentic teammates” are used interchangeably) [1]. While current AI tools are powerful, they are passive: a user has to initiate every interaction, provide all context, and interpret all outputs. In comparison, autonomous AI agents act without prompting, perform sequential steps independently, gather information from multiple sources, collaborate with humans or other agents, and self-improve through feedback loops [1]. Essentially, AI agents can behave as teammates with assigned roles and responsibilities. Of note, agentic AI is distinct from traditional rule-based automation, which executes fixed logic on structured inputs. In contrast, AI agents use large language models (LLMs) to reason across unstructured data and handle novel situations, which go beyond what deterministic automation can achieve.

Large health care systems have already begun leveraging agentic AI to reduce administrative burden and improve quality of care. For example, Duke Health intends to deploy AI agents in cardiology for care coordination that autonomously handle scheduling requests, optimize resource allocation, and connect patients to clinical trials [2]. At Ochsner Health, AI agents continuously review patient panels of primary care providers, reaching out to patients for screenings, lab monitoring, and postdischarge care coordination [3]. Oxford University Hospitals is piloting AI agents in oncology that summarize charts, determine tumor staging, and draft guideline-compliant plans for multidisciplinary tumor boards [4]. These early implementations demonstrate the feasibility of agentic AI across multiple specialties and clinical settings.

## Opportunities and Risks for Dermatology

Dermatology is particularly well-positioned to benefit from these emerging AI capabilities given its high patient volumes, substantial administrative burdens, and clinicopathological diagnostic approach [5]. In a future dermatology practice, AI agents could tackle high patient volumes through intelligent scheduling (eg, spacing out procedures and complex visits), previsit charting, scribing and coding, and automated patient follow-up. Voice agents can reach even more patients through phone calls, including older adults and patients with limited English proficiency. AI agents can identify and recruit patients for clinical trials, particularly to address the critical need for diverse representation. Medication access (eg, prior authorization, appeals, patient assistance programs, and laboratory monitoring) for specialty drugs like biologics is also well-suited for an agentic workflow that eases the burden of manual labor on support staff. The unique clinicopathological workflow in dermatology also lends itself to multistep agentic coordination: an agentic system could receive a pathology result, verify the correct patient and anatomic site against prior documentation and images, alert the dermatologist, and coordinate definitive treatment (eg, reaching out to a Mohs surgeon).

Despite the promising applications, there are limited published case studies of agentic AI in the dermatology literature. Nonetheless, there are numerous companies that have been launched in the past few years that are targeting every aspect of the dermatology workflow with an agentic approach. As health care systems and other specialties begin establishing the workflows, norms, and business models for agentic AI, dermatologists must actively educate themselves on this technology and engage to shape its real-world implementation.

**Figure 1.**
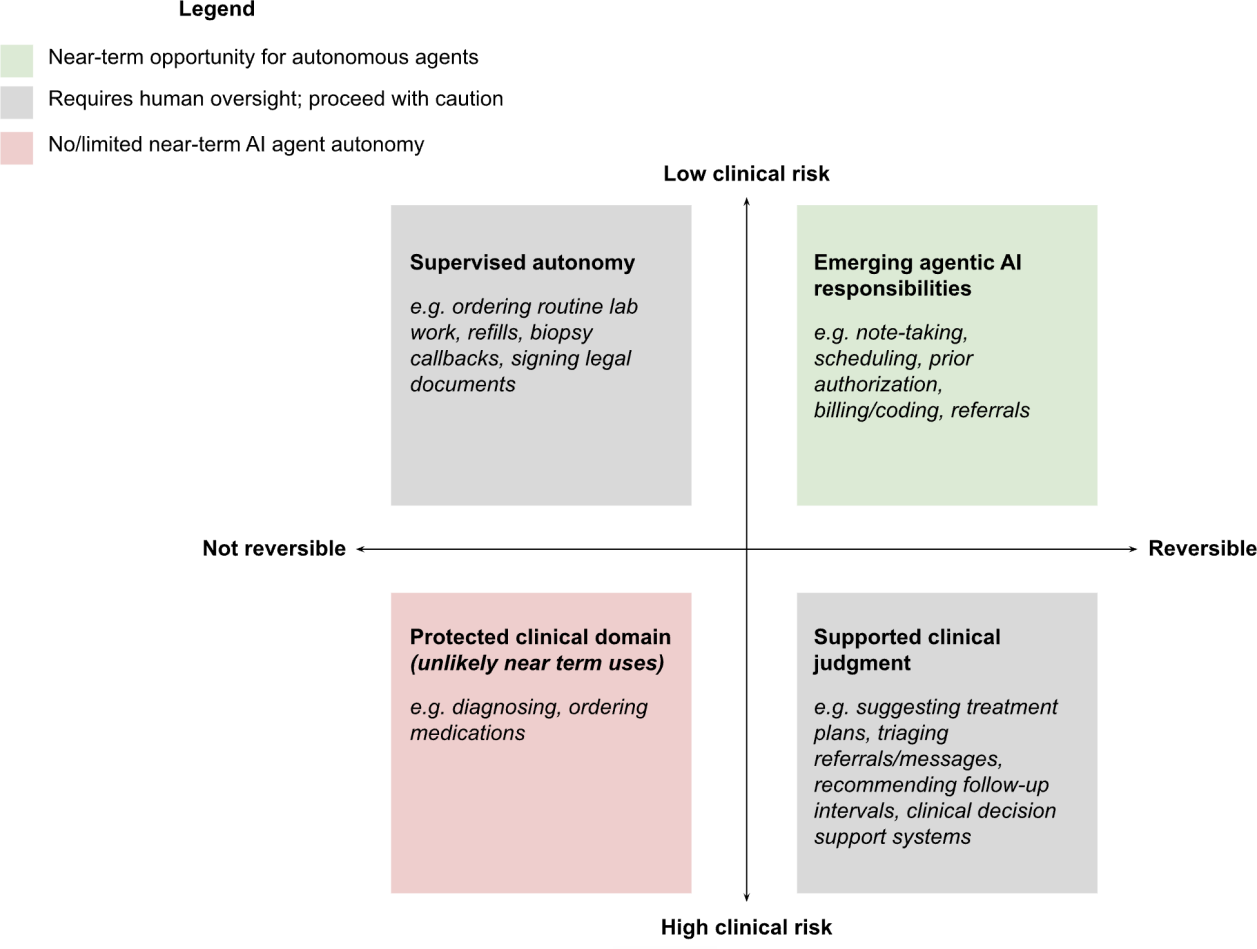
Autonomous agentic AI is most ready for tasks that are reversible and of low clinical risk. When tasks are irreversible or of high clinical risk, stronger oversight with a human dermatologist is warranted. Tasks that are both irreversible and of high clinical risk (eg, diagnosing, ordering medications) are unlikely to be ready for near-term adoption and should have limited AI agent autonomy. The placement of example tasks within this framework is intended as a general guide, and exact positioning varies depending on institutional workflows, local staffing models, or electronic health record (EHR) capabilities.

Deploying agentic AI requires balancing risk; when considering pilot implementations, dermatologists should take a graduated approach for delegating tasks to autonomous agents. To assess readiness for agentic AI in dermatology practices, we propose the following framework (Figure 1): promising agentic applications ready for pilots focus on reversible, low-risk tasks. Tasks that are irreversible or carry higher clinical risk warrant stronger oversight by dermatologists or staff and therefore require additional caution before fully adopting agentic teammates. High-stakes, irreversible decisions with major clinical risk are not suitable for delegation to AI agents in near-term, real-world settings.

Significant barriers also impede the widespread deployment of AI agents in dermatology. First, current AI models are not ready for the reliability and complexity necessary in real-world clinical settings. A recent study demonstrated that the best-performing LLMs successfully completed 70% of representative clinical tasks, such as medication and test ordering, referrals, documentation, and patient communication. Models struggled most on tasks that required modifying records rather than simply retrieving information, highlighting the need for stepwise adoption [6]. Second, interoperability must be improved so that agents can gather data from siloed health records. Even with full access, agents need the right permissions to actually take action on behalf of dermatologists. Third, fundamental questions need to be addressed about liability, privacy, and regulation, as historically, regulatory agencies judge an AI-enabled medical device for a specific task, rather than a broad range of interconnected tasks [1,7].

## Why Dermatologists Must Lead Now

Though this technology may seem distant for the practicing dermatologist, decisions about agentic AI are being made now. National and international dermatology organizations should include agentic AI on their agendas for AI use, seek partnerships with companies, and write guidelines for deploying AI agents. Unlike previous AI guidance focused on diagnostic performance, agentic AI guidelines must address multistep reasoning, minimum performance standards, mechanisms requiring human oversight, and protocols for monitoring ongoing safety. Concrete near-term actions include establishing a dedicated agentic AI task force within professional societies such as the American Academy of Dermatology, developing standardized reporting criteria for pilot programs in dermatology practices, and creating a voluntary registry of deployments to facilitate shared learning.

Research should also specifically examine agent performance across diverse patient populations to mitigate bias [7]. Although there are well-documented performance disparities of *diagnostic* AI tools on darker skin tones, it is unclear whether agentic AI systems that focus on nondiagnostic tasks will face the same challenges. However, agentic AI systems that rely on flawed components risk amplifying these disparities at scale, making them invisible in automated workflows. Safeguards should include ongoing postdeployment monitoring across demographic subgroups.

Agentic AI reflects an emerging paradigm where intelligent, autonomous agents act on behalf of physicians and patients, enhancing efficiency, continuity, and patient experience. There is no substitute for the human physician–patient relationship, and agentic AI offers dermatologists an opportunity to strengthen this relationship, not replace it. Dermatologists must not only stay informed of advances in agentic AI; we must also actively shape decisions about agentic AI to ensure appropriate, ethical, and optimized deployment to reflect our values and patients’ needs.

## Abbreviations

AI: artificial intelligence
LLM: large language model
